# Comparison of the analytical and clinical sensitivities of 34 rapid antigen tests with prevalent SARS-CoV-2 variants of concern during the COVID-19 pandemic in the UK

**DOI:** 10.1128/spectrum.00749-25

**Published:** 2025-08-13

**Authors:** Rachel L. Byrne, Rachel S. Owen, Ghaith Aljayyoussi, Caitlin Greenland-Bews, Konstantina Kontogianni, Anushri Somasundaran, Dominic Wooding, Christopher T. Williams, LSTM Diagnostics Group, Kate Buist, Falcon Steering Group, A. Joy Allen, Margaretha de Vos, Richard Body, Emily R. Adams, Camille Escadafal, Thomas Edwards, Ana I. Cubas-Atienzar

**Affiliations:** 1 Centre for Drugs and Diagnostics, Liverpool School of Tropical Medicine 9655, Liverpool, United Kingdom; 2 FIND, Foundation for Innovative New Diagnostics 91635, Geneva, Switzerland; 3 Manchester University NHS Foundation Trust 5293, Manchester, United Kingdom; Victorian Infectious Diseases Reference Laboratory, Melbourne, Australia

**Keywords:** rapid diagnostic test (RDT), antigen detection, COVID-19, SARS-CoV-2 variant of concern (VOC), B.1.617.2, Delta, B.1.1.529, Omicron, limit of detection (LOD)

## Abstract

Antigen-detection rapid diagnostic tests (Ag-RDTs) have become a central pillar for the management of coronavirus disease worldwide due to their speed and ease of use and are now being developed for use in other emerging outbreaks. Like other viruses, severe acute respiratory syndrome coronavirus 2 (SARS-CoV-2) is subject to rapid mutation as it spreads, and new variants of concern (VOCs) emerge frequently, posing a significant challenge for the detection of newer, highly mutated variants. It is, therefore, important that the performance of Ag-RDTs is regularly evaluated, particularly in outbreak scenarios where rapid diagnostics are key to limiting disease spread. Here, we present a comprehensive evaluation of the analytical and clinical sensitivities of 34 commercially available Ag-RDTs with five SARS-CoV-2 VOCs, all of which were highly prevalent in the UK at various times between 2019 and 2023. This study highlights the importance of regular evaluation of the Ag-RDT performance, with several Ag-RDTs demonstrating a reduced performance with some VOCs. We conclude that a regular performance evaluation through our proposed pipeline, combined with a broad consensus approval threshold across global organizations, is essential to maintaining the effectiveness of Ag-RDTs as a disease management tool during outbreaks.

**IMPORTANCE:**

Antigen-detection rapid diagnostic tests (Ag-RDTs) came to global prominence during the coronavirus disease pandemic, where they offered a quick and simple at-home diagnostic, which could be used to manage disease spread. A major ongoing challenge for the broad use of Ag-RDTs is the speed at which new SARS-CoV-2 variants emerge, each of which has the potential to reduce the performance of available Ag-RDTs. As Ag-RDTs are explored for use in other viral disease outbreaks, pipelines for the regular evaluation of test performance are essential for ensuring that Ag-RDTs can be employed effectively. Here, we have developed a robust pipeline for the large-scale evaluation of commercially available Ag-RDTs against several major SARS-CoV-2 variants, which can be adapted and applied to other emerging outbreaks to ensure that test performance is maintained as a virus evolves.

## INTRODUCTION

Antigen-detection rapid diagnostic tests (Ag-RDTs) offer quick, inexpensive, laboratory-independent diagnostics that can be performed at home by lay individuals (1, 2). The emergence of severe acute respiratory syndrome coronavirus 2 (SARS-CoV-2) in late 2019 and the subsequent coronavirus disease (COVID-19) pandemic resulted in significant global health challenges and financial losses (3), and given the importance of early detection and isolation in disease management (4), Ag-RDTs became a central pillar of control strategies and are now the first-line diagnostic in many countries (5, 6). While the development and deployment of SARS-CoV-2 Ag-RDTs were rapid, the circulating virus accumulated mutations resulting in the emergence of new viral strains with characteristics that posed a threat to current treatment and diagnostic options (variants of concern [VOCs]). The frequent emergence of new VOCs, combined with the global nature of the pandemic response, posed a continuous challenge to disease monitoring (6). The majority of Ag-RDTs were developed and evaluated early in the COVID-19 pandemic, utilizing the Ancestral (WT) SARS-CoV-2’s nucleocapsid (N) protein as a target due to its high abundance within the virion (7). However, VOCs rapidly emerged, including Alpha, Beta, Gamma, and Delta, and, by the time of the emergence of the first Omicron VOC in late 2021, the viral genome had accumulated a significant number of mutations (8, 9). These mutations included several within the N protein, where the Omicron lineage has three unique mutations, making it difficult to predict the performance of Ag-RDTs with emerging VOCs (10).

Initial data on the performance of Ag-RDTs for different COVID-19 VOCs remain contradictory in both analytical and clinical evaluations. While early reports evaluating a small number of brands found comparable sensitivities between WT, Delta, and Omicron (B.1.1.529) VOCs (11–13), later studies demonstrated that Ag-RDTs showed loss of sensitivity to Omicron when compared to other VOCs (14, 15), and clinical evaluations of Ag-RDTs against emerging SARS-CoV-2 lineages have reported inconsistent results (14, 16, 17).

For Ag-RDTs to be used to their full potential in managing large-scale disease outbreaks, there must be confidence that these tests maintain good clinical specificity, even as a pathogen evolves, and regular clinical and analytical evaluations are essential to ensuring that Ag-RDTs are performing to the required standards. Given the comparatively recent emergence of Ag-RDT testing as a front-line disease control measure, relatively few evaluations of these devices on a large scale over time have been conducted. The aim of this study was to carry out a comprehensive evaluation of the performance of 34 commercially available Ag-RDTs against SARS-CoV-2 using the most prevalent VOCs in the United Kingdom between 2019 and 2023, and we present herein a robust pipeline for the large-scale evaluation of rapid diagnostic tests as new variants emerge.

## RESULTS

### Analytical sensitivity using cultured SARS-CoV-2 virus

**Fig 1:**
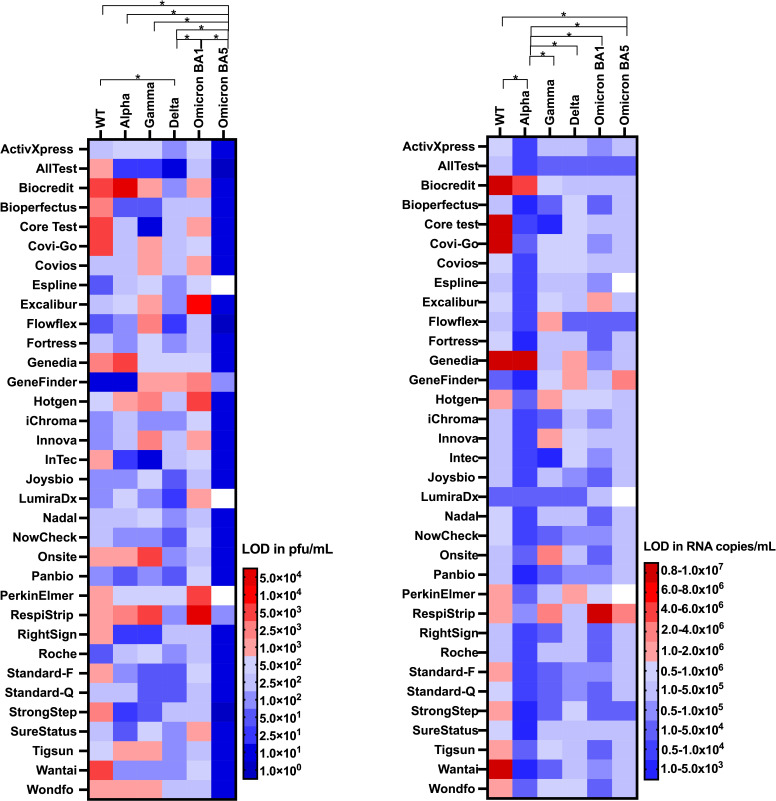
Heatmap comparing the LODs of 34 Ag-RDTs using the Ancestral (WT), Alpha (B.1.1.7), Gamma (P.1), Delta (B.1.617.2) Omicron (BA.1), and Omicron (BA.5) variants on PFU/mL (left) and RNA copies/mL (right). Ag-RDT brands are given on the *y* axis, while the SARS-CoV-2 strain is given on the *x* axis. The blue colors indicate LODs fulfilling the DHSC (for PFU/mL) and WHO criteria (for RNA copies/mL), while the red colors indicate LODs that fail to meet these criteria * = *P* ≤ .05 between VOCs. Data of the Ancestral, Alpha, and Gamma have been taken from our previously published work (21).

The analytical sensitivity and the limit of detection (LOD) of 34 SARS-CoV-2 Ag-RDTs were evaluated using viral cell cultures quantified by plaque assays (PFU/mL) and RT-qPCR (RNA copies/mL). For Omicron sub-lineage BA.5, all of the 34 Ag-RDTs evaluated had an LOD ≤ 5.0 × 10^2^ PFU/mL, fulfilling the criteria set by the British Department of Health and Social Care (DHSC) (Fig. 1), and all, except two brands (RespiStrip and GeneFinder), had an LOD ≤ 1.0 × 10^6^ RNA copies/mL, thus fulfilling the World Health Organization (WHO) and the UK Medicines and Healthcare Products Regulatory Agency (MHRA) Target Product Profile (TPP) recommendations for SARS-CoV-2 Ag-RDTs (18, 19). In contrast, for Omicron sub-lineage BA.1, only 23 of the 34 Ag-RDTs evaluated had an analytical LOD ≤ 5.0 × 10^2^ PFU/mL, failing to fulfill the DHSC criteria. Despite this, 32 out of 34 (including Biocredit, Core, Covios, Hotgen, Innova, LumiraDx, PerkinElmer, and SureStatus, that all fell below the DHSC recommendations) had an LOD ≤ 1.0 × 10^6^ RNA copies/mL, fulfilling WHO and MHRA criteria. AllTest, Bioperfectus, Flowflex, Fortress, Joysbio, Nadal, Onsite, RightSign, Roche, StrongStep, Standard Q, Tingsun, and Wondfo were the more sensitive Ag-RDTs for Omicron BA.1 (see Fig. 1, left and right) with an LOD ≤ 2.5 × 10^2^ PFU/mL and 4.4 × 10^4^ RNA copies/mL. For both Omicron BA.1 and BA.5, the Ag-RDT brand with the lowest sensitivity was RespiStrip with an LOD of 5.0 × 10^4^ PFU/mL and 9.2 × 10^6^ RNA copies/mL (BA.1) and LOD of 1.0 × 10^2^ PFU/mL and 3.5 × 10^6^ RNA copies/mL (BA.5), respectively.

For the Delta VOC, 33 out of the 34 Ag-RDTs evaluated had an LOD ≤ 5.0 × 10^2^ PFU/mL, and 31 of the 34 reported an LOD ≤ 1.0 × 10^6^ RNA copies/mL (Fig. 1), with GeneFinder failing to meet either the DHSC- or WHO-recommended LOD.

For the Alpha VOC, 27 of the 34 Ag-RDTs evaluated had an analytical LOD ≤ 5.0 × 10^2^ PFU/mL, with Biocredit, Genedia, Hotgen, Onsite, RespiStrip, Tigsun, and Wondfo failing to meet the DHSC minimum requirements. Biocredit and Genedia also fell below the WHO requirement of 1.0 × 10^6^ RNA copies/mL.

For the Gamma VOC, five Ag-RDT brands (Flowflex, Hotgen, Innova, Onsite, and RespiStrip) failed to meet either the DHSC or WHO requirements with a further seven Ag-RDT brands falling below the DHSC recommendations. The Ag-RDTs with the greatest sensitivity for Gamma VOC were AllTest, Core Test, InTec, Standard-F, Standard-Q, StrongStep, and Surestatus (Fig. 1, left).

For the WT, the target for which all Ag-RDT brands were originally developed, only 19 and 22 met the DHSC and WHO requirements, respectively.

When comparing only the PFU/mL values, we found that tests had significantly higher LODs with Omicron BA.1 compared to Delta (*P* = 0.000) and significantly lower LODs with Omicron BA.5 compared to all other VOCs tested (*P* = 0.001). When comparing RNA copies/mL, the Ag-RDTs detected Alpha VOC (*P* = 0.000) more sensitively than the other VOCs (*P* = 0.000).

### Retrospective samples: SARS-CoV-2 Ag-RDT clinical sensitivity

**Fig 2:**
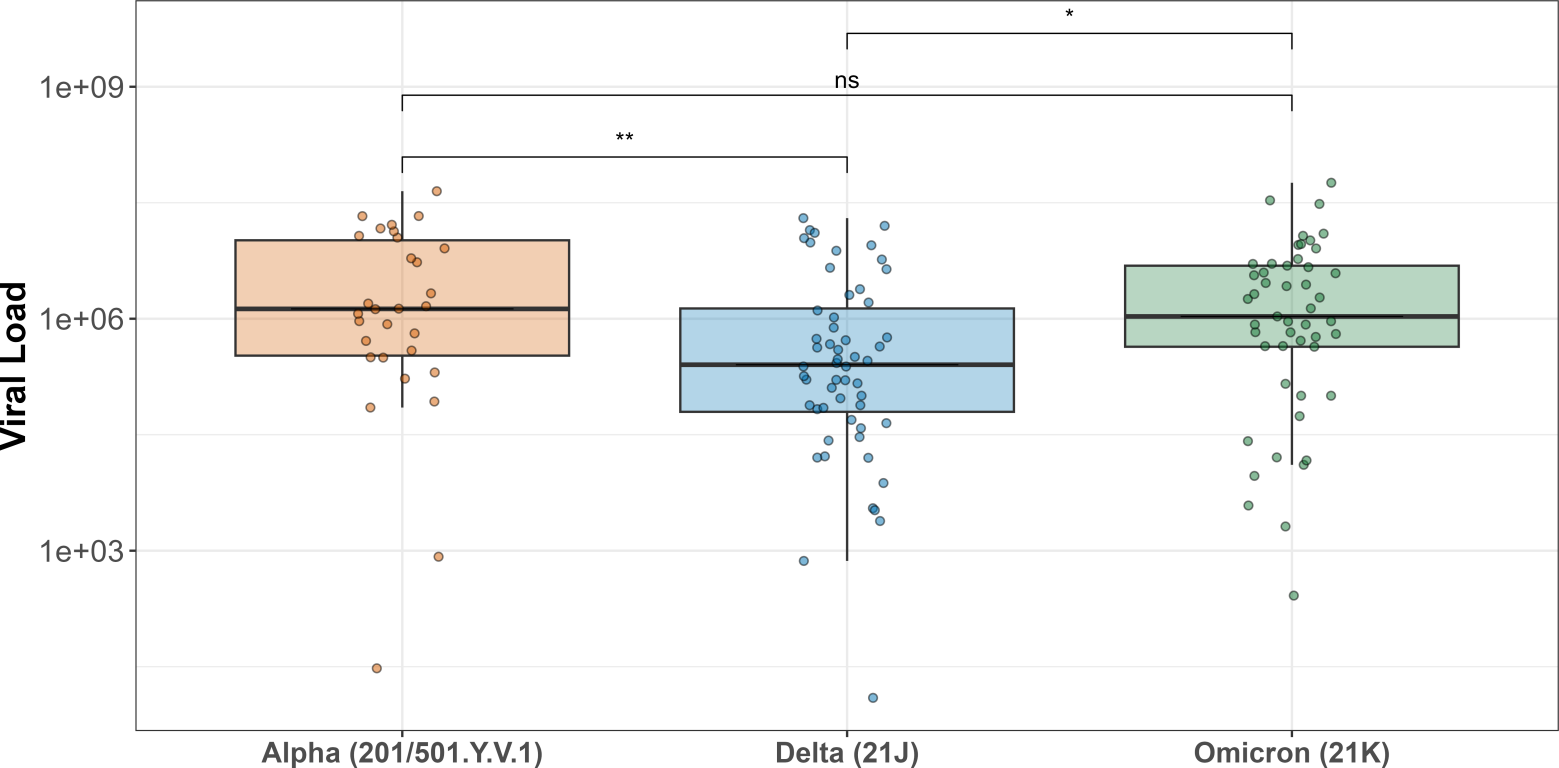
Boxplot of the SARS-CoV-2 viral load (RNA copies/mL) as quantified by RT-qPCR on the NP swabs collected from participants recruited to FALCON between January 2021 and March 2022. Whiskers indicate 95% confidence intervals, and the horizontal line indicates the median. Asterisks indicate the statistical significance between different VOCs as determined by the Kruskal-Wallis test (ns = non-significant, * = *P* ≤ .05, ** = *P* ≤ .01)

The clinical sensitivity of five Ag-RDT brands (Covios, Flowflex, Hotgen, Onsite, and SureStatus) was evaluated utilizing SARS-CoV-2 Alpha (*n* = 30)-, Delta (*n* = 56)-, and Omicron (*n* = 49)-positive nasopharyngeal (NP) swabs in viral transport medium (VTM) stored at −80°C. The viral load in clinical samples was determined using the COVID-19 Genesig RT-qPCR Kit as a gold standard reference. Statistically higher viral loads determined by RT-qPCR were recorded among individuals positive for Alpha and Omicron infections compared to Delta (*P* = 0.001 and *P* = 0.009, respectively) (Fig. 2).

**Fig 3:**
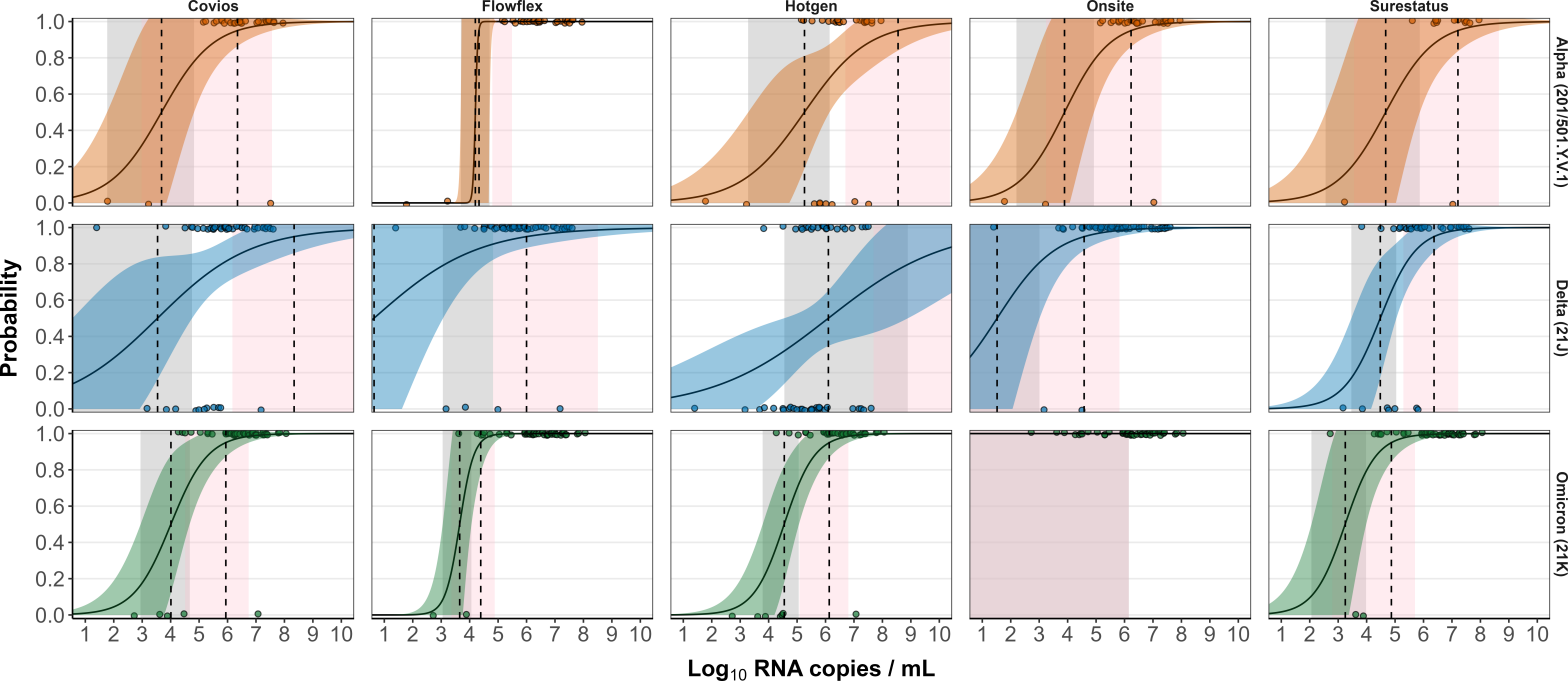
Limit of detection analyses of the upper-respiratory samples positive by RT-qPCR for five SARS-CoV-2 Ag-RDT tests (Covios, Hotgen, Onsite, Flowflex, and SureStatus) using NP swabs. The log10 RNA copies on the *x* axis were plotted against a positive (1.0) or negative (0.0) Ag-RDT result on the *y* axis. Fill curves show the logistic regressions of the viral load on the Ag-RDT result; the vertical dashed lines indicate the log10 RNA copies subjected to the test at which 50 and 95% LOD of the samples are expected to be positive based on the regression results. No significant differences were observed.

We determined the 50 and 95% LODs with Alpha, Delta, and Omicron SARS-CoV-2-positive swab samples for five Ag-RDT brands based on a logistic regression model (Table 1, Fig. 3). Overall, the lowest LOD for the Alpha VOC was recorded with Flowflex Ag-RDT (50% LOD 1.58 × 10^4^ RNA copies/mL and 95% LOD 2.14 × 10^4^ RNA copies/mL), for Delta variant with Onsite Ag-RDT (50% LOD 3.31 × 10^1^ RNA copies/mL and 95% LOD 3.80 × 10^4^ RNA copies/mL), and for Omicron with SureStatus Ag-RDT (50% LOD 1.78 × 10^3^ RNA copies/mL and 95% LOD 7.41 × 10^4^ RNA copies/mL), which were all statistically similar to the reported analytical LOD. The Delta variant exhibited the greatest variability between the predicted LODs from different Ag-RDTs (Table 1), whereas for the analytical LOD, the Alpha VOC showed the greatest variability (Fig. 1).

**TABLE 1:** 50 and 95% limits of detection (LOD) (RNA copies/mL) for five Ag-RDT brands (Covios, Flowflex, Hotgen, Onsite, and SureStatus) from 122 clinical NP samples positive for Alpha, Delta, and Omicron SARS-CoV-2 VOCs^*a*^

		Ag-RDT brand (RNA copies/mL)				
	LOD	Covios	Flowflex	Hotgen	Onsite	SureStatus
Alpha	50%	4.79E + 03	1.58E + 04	1.82E + 05	7.76E + 03	4.68E + 04
	95%	2.24E + 06	2.14E + 04	3.55E + 08	1.70E + 06	1.62E + 07
Delta	50%	3.47E + 03	4.37E + 00	1.26E + 06	3.31E + 01	3.02E + 04
	95%	2.19E + 08	1.00E + 06	1.00E + 12	3.80E + 04	2.34E + 06
Omicron	50%	1.02E + 04	4.47E + 03	3.55E + 04	0	1.78E + 03
	95%	8.71E + 05	2.45E + 04	1.35E + 06	0	7.41E + 04

^*a*^ LOD is calculated from the RT-qPCR quantification of the viral loads presented in Fig. 2.

**Fig 4:**
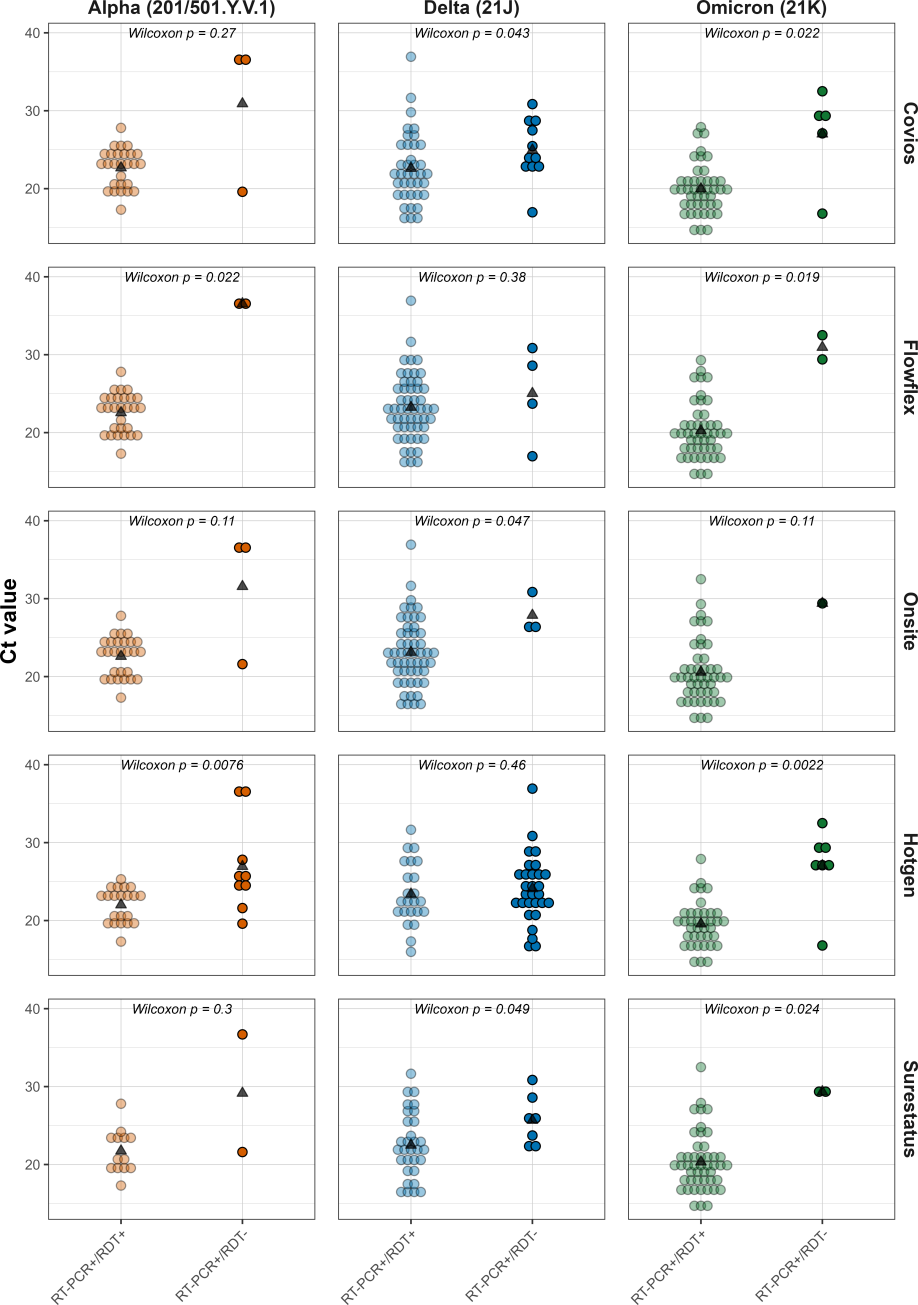
Positive and negative Ag-RDT results according to the RT-qPCR cycle threshold (Ct) values. The *P*-values indicate the results of the Bonferroni-adjusted post-hoc tests performed following three-way factorial ANOVA to compare Ag-RDT+ and Ag-RDT− within each variant and test brand. Asterisks indicate comparisons where Cohen’s *d* ≥ 0.5 and *P* < 0.05, indicating a significant difference between the genomic Ct values of RT-qPCR+/Ag-RDT+ and RT-qPCR+/Ag-RDT− samples.

All Ag-RDTs, with the exception of Hotgen, satisfy the WHO and UK MHRA minimum sensitivity requirement of 80% against Alpha VOC, with the clinical sensitivities ranging from 70 to 93.3% (Hotgen and Flowflex, respectively) (Table 2). For Delta VOC, three of the Ag-RDTs (Flowflex, Onsite, and Surestatus) met the minimum sensitivity requirement of 80%, with Covios falling marginally short at 77.6% and Hotgen reporting a significantly lower sensitivity of 44.2%. The observed sensitivities for Omicron were consistent across all brands of Ag-RDTs from 84.4 to 97.9% (Hotgen and Onsite), all satisfying the minimum requirements. For samples with low Ct values (<25), the sensitivities were statistically similar for the Covios, Flowflex, Onsite, and SureStatus Ag-RDTs but not for the Hotgen Ag-RDT. For the Hotgen Ag-RDT, the sensitivity for the Delta VOC was significantly lower compared to Omicron (*P* < 0.001) and Alpha (*P* = 0.011). High Ct values (Ct > 25) resulted in reduced test sensitivities across all variants, with the greatest sensitivity reported in samples positive with the Omicron VOC. A three-way factorial analysis of variance (ANOVA) assessing the effects of the RDT result (value: positive vs. negative), variant, and test brand, along with their interactions, revealed a significant main effect of the Ag-RDT result (*F*[1, 593] = 98.21, *P* < 0.001, η²*P* = 0.14), indicating a large effect size (Fig. 4). The effect of variant was also significant (*F*[2, 593] = 27.83, *P* < 0.001, η²*P* = 0.09), while test brand had a small and non-significant effect (*F*[4, 593] = 1.58, *P* = 0.18, η²*P* = 0.01). The interaction between the RDT result and variant was significant (*F*[2, 593] = 14.20, *P* < 0.001, η²*P* = 0.05), suggesting that the effect of genomic Ct on the RDT result differs across variants. Other interactions, including the three-way interaction, showed negligible effect sizes (η²*P* < 0.01) and were not significant.

**TABLE 2:** Comparison of the clinical sensitivities of the five antigen rapid diagnostic tests (Ag-RDTs) between the Alpha, Delta, and Omicron variants using retrospective clinical samples^*a*^

Population	Variant	Covios	Flowflex	Onsite	Hotgen	Surestatus
Total cohort	Alpha (CI 95%)	90%, 30 73.5–97.9%	93.3%, 30 77.9–99.2%	90%, 30 73.5–97.9%	70%, 30 50.6–85.3%	86.7%, 15 59.5–98.3%
	Delta (CI 95%)	77.6%, 49 63.4–88.2%	92.9%, 56 82.7–98%	94.5%, 55 84.9–98.9%	44.2%, 52 30.5–58.7%	81.6%, 38 65.7–92.3%
	Omicron (CI 95%)	89.8%, 49 77.8–96.6%	95.8%, 48 85.7–99.5%	97.9%, 47 88.7–99.9%	84.4%, 45 70.5–93.5%	95.9%, 49 86–99.5%
	All (CI 95%)	85.2%, 128 77.8–90.8%	94%, 134 88.6–97.4%	94.7%, 132 89.4–97.8%	64.6%, 127 55.6–72.8%	89.2%, 102 81.5–94.5%
≤Ct 25	Alpha (CI 95%)	95.8%, 24 78.9–99.9%	100%, 24 85.8–100%	95.8%, 24 78.9–99.9%	83.3%, 24 62.6–95.3%	92.3%, 13 64–99.8%
	Delta (CI 95%)	81.8%, 33 64.5–93%	94.6%, 37 81.8–99.3%	100%, 36 90.3–100%	45.5%, 33 28.1–63.6%	88%, 25 68.8–97.5%
	Omicron (CI 95%)	97.6%, 42 87.4–99.9%	100%, 41 91.4–100%	100%, 40 91.2–100%	97.4%, 38 86.2–99.9%	100%, 42 91.6–100%
	All (CI 95%)	91.9%, 99 84.7–96.4%	98%, 102 93.1–99.8%	99%, 100 94.6–100%	75.8%, 95 65.9–84%	95%, 80 87.7–98.6%
> Ct 25	Alpha (CI 95%)	66.7%, 6 22.3–95.7%	66.7%, 6 22.3–95.7%	66.7%, 6 22.3–95.7%	16.7%, 6 0.4–64.1%	50%, 2 1.3–98.7%
	Delta (CI 95%)	68.8%, 16 41.3–89%	89.5%, 19 66.9–98.7%	84.2%, 19 60.4–96.6%	42.1%, 19 20.3–66.5%	69.2%, 13 38.6–90.9%
	Omicron (CI 95%)	42.9%, 7 9.9–81.6%	71.4%, 7 29–96.3%	85.7%, 7 42.1–99.6%	14.3%, 7 0.4–57.9%	71.4%, 7 29–96.3%
	All (CI 95%)	62.1%, 29 42.3–79.3%	81.2%, 32 63.6–92.8%	81.2%, 32 63.6–92.8%	31.2%, 32 16.1–50%	68.2%, 22 45.1–86.1%

^*a*^ Calculated sensitivities, sample size (in italics), and 95% confidence intervals (CI 95%) are given. Ct = cycle threshold.

### Prospective samples: SARS-CoV-2 Ag-RDT clinical sensitivity

During the prospective evaluation, 122 participants tested positive by RT-qPCR for SARS-CoV-2. Of them, 32 were the Delta VOC, and 90 were Omicron (BA.1) VOC. Samples were immediately tested using the Biocredit (RapiGEN, South Korea) Ag-RDT, with 99 yielding a positive Biocredit Ag-RDT result (sensitivity 81.1%, confidence interval [CI] 95% 73.1–87.6%), satisfying the minimum clinical sensitivity requirements. When separated by VOC, the sensitivity for Delta VOC dropped below these requirements (71.9% [CI 95% 53.3–86.6%]), although this was statistically similar to the acceptable sensitivity observed for Omicron VOC (84.4% [CI 95% 75.3–91.2%)] and may be due to the lower sample size. The difference in the Ct value between positive and negative samples by Biocredit was significant (Delta *P* = 0.038, Omicron *P* = 0.00007) across both VOCs. Additionally, the intensity of the test band of positive Ag-RDTs was recorded for 119 samples (three excluded due to line intensity not recorded), with no significance obtained between the two VOCs (Delta and Omicron BA.1).

## DISCUSSION

The rapid emergence of new VOCs throughout the course of the COVID-19 pandemic posed a significant challenge for reliable rapid diagnostic testing (16). New VOCs often contained mutations in the N gene, increasing the likelihood that RDT analytical sensitivity could be compromised. Indeed, studies have identified mutations in the N gene which affected the sensitivity of Ag-RDTs, including the T135I mutation in the Alpha VOC (20), and the A376T and M241I mutations (21), thus it is important to maintain regular assessment of Ag-RDT performance over time. Ag-RDTs, which have been developed for other viral illnesses, particularly for other respiratory viruses such as influenza viruses and respiratory syncytial virus, suffer from similar potential performance problems as the viruses mutate and new strains emerge, causing highly variable clinical performance and further highlighting the need for continuous evaluation of rapid testing devices (22–24). We present a comprehensive assessment of the analytical sensitivity of 34 commercially available COVID-19 Ag-RDTs for the detection of the Omicron (BA.1 and BA.5), Alpha, Gamma, and Delta VOCs and WT, the major variants circulating in the United Kingdom between 2019 and 2023 (8).

Analytically, the majority of Ag-RDTs successfully met the WHO criteria outlined in the TPP for SARS-CoV-2 Ag-RDTs when detecting the Omicron viral lineages (94.1%, BA.1 and 93.5%, BA.5) (18). When analyzing RNA copies/mL, as outlined in the WHO recommendations, the LOD of Omicron VOC BA.5 was statistically lower than that of the WT and Alpha VOCs but similar to the Gamma, Delta, and Omicron BA.1 VOCs. However, when comparing the PFU/mL, recommended by the UK DHSC, tests have significantly lower LODs with Omicron BA.5 than all other VOCs and WT. The sensitivity of the Ag-RDTs for Omicron VOC determined during this study was found to be higher than some other studies have previously determined (25, 26). The discrepancy in sensitivities with previous studies could be due to different experimental designs or variations in Ag-RDT batch or brand performance; however, as the data presented here were generated in the same way, we believe this presents a robust and useful comparison across variants and test brands for determining the performance of Ag-RDTs relative to one another. The observed discrepancies between RNA copies/mL and PFU/mL have been reported in previous studies (27–30) and have been attributed to differences in the virus’ ability to form plaques and varying ratios of infectious particles to RNA copies present (31). While the PFU/mL measures infectious virus present, RNA copies/mL encompasses all SARS-CoV-2 RNA present, including non-infectious or dead virus. For the Ag-RDT evaluation, RNA copies/mL is recommended by the WHO and UK MHRA, and the UK DHSC recommends PFU/mL. It is counterintuitive for organizations to utilize different units of measurements for the comparison of Ag-RDTs, and a consensus would allow a more appropriate evaluation, something that is essential for effectively managing responses to new disease outbreaks.

For five of the 34 Ag-RDT brands, we present clinical accuracy data utilizing clinical NP samples positive for SARS-CoV-2 Alpha, Delta, and Omicron (BA.1) VOCs. Hotgen consistently performed poorer than other Ag-RDTs, typically falling below the WHO and UK MHRA TPP guidance for clinical sensitivity, with the exception of the detection of Omicron VOC (18, 32). For all brands, the clinical sensitivity values obtained with retrospective samples were the highest for the Omicron samples. A similar trend was observed when using only prospective samples collected when the UK was experiencing the Delta and Omicron waves of infection. A lower sensitivity was recorded for samples positive for the Delta VOC compared to those positive with an Omicron VOC infection on the Biocredit Ag-RDT, consistent with the analytical data published by Stanley et al. (33). Previous studies for determining the performance of Ag-RDTs during the emergence of the Omicron VOC yielded varying results, with some studies reporting reductions in sensitivity (16, 34, 35) and others reporting similar or improved Ag-RDT sensitivity for Omicron VOC (36, 37). These discrepancies may be due to the sample collection method (NP vs oropharyngeal [OP] swabs) or due to the symptom status of recruited patients, both of which have been shown to impact SARS-CoV-2 detection (38, 39). To reduce the variation introduced by these factors, this study only uses NP swabs collected from symptomatic adults for both the prospective and retrospective clinical studies. The differences in sensitivity of Ag-RDTs between VOCs, which are independent of the temporal emergence of each variant, highlight the importance of a continual and thorough assessment of the RDT performance as new lineages of a pathogen emerge.

For all brands in the retrospective cohort, the predicted clinical LODs were statistically similar to the analytical LODs obtained with spiked laboratory samples. Clinical evaluation is expensive, with surplus samples often not available for comprehensive testing, hindering the large-scale assessment of the Ag-RDT performance. The data presented here demonstrate the efficacy of analytical samples, indicating that analytical evaluation is a robust alternative for evaluating the Ag-RDT performance in the absence of abundant clinical samples. Practically, this provides confidence that the LOD of Ag-RDTs can be determined from analytical samples in the context of emerging outbreaks, where significant numbers of clinical samples may not be readily available for testing. Analytical testing can instead be rapidly implemented in these scenarios to quickly determine the likelihood that a newly emerged VOC will reduce the Ag-RDT performance, streamlining the evaluation process to ensure tests can be updated when necessary. Further validation of this correlation using fresh prospective clinical samples would strengthen confidence in using analytical LODs as an indicator of clinical performance.

This study has several strengths; we have carried out an extensive evaluation of the analytical sensitivity of 34 commercially available Ag-RDT brands. This list is inclusive of most WHO-EUL-recommended tests and five awaiting approvals at the time of the study, thus of high global public health relevance. Additionally, we included both viral isolates and clinical specimens to evaluate the Ag-RDT sensitivity. The clinical specimens used in this study are attributed to three different lineages: Alpha, Delta, and Omicron, which were all at some point a dominant VOC, providing a comprehensive evaluation of assay performance across a significant temporal and mutational timeframe.

A limitation of this study is the use of retrospective frozen specimens instead of fresh swabs as recommended by most Ag-RDT manufacturers, which may reduce test sensitivity. However, prospective clinical evaluation studies rarely include multiple VOCs, as their prevalence depends on their time period, and the prospective evaluation of multiple RDT brands simultaneously is complicated by the need for a single swab per test. To correct for the potential degradation of RNA after a freeze-thaw cycle, viral RNA was re-tested by RT-PCR at the time of Ag-RDT evaluation, and these values were used for comparison.

To conclude, we present a robust pipeline for the large-scale evaluation of commercially available Ag-RDTs against multiple SARS-CoV-2 VOCs, which can be easily and rapidly deployed to assess further variants or for different disease outbreaks. We report similar, if not superior, LODs for Omicron compared to other VOCs and WT across a wide range of commercially available RDTs. However, we also report decreased detection of the Delta VOC in both analytical and clinical samples, highlighting the need for continuous assessment of Ag-RDTs, especially those recommended for at-home testing. We additionally report inconsistencies between product fulfillment criteria for WHO and UK DHSC, where several tests show significantly different performances depending on the guidelines, highlighting the importance of standardized evaluation criteria for Ag-RDTs, particularly during a global response. Finally, we demonstrate that analytical sensitivity can be used to predict reduction in the clinical sensitivity of Ag-RDTs following the emergence of a new VOC. This allows for the rapid assessment of test performance during an outbreak setting, even in the absence of large numbers of clinical samples, improving the ability of frontline clinicians and test developers to respond to emerging VOCs.

## MATERIALS AND METHODS

### Evaluated Ag-RDTs

Thirty-four Ag-RDT brands were evaluated in this study; all were lateral flow assays (LFA), among which 31 use colorimetric gold nanoparticle detection; two use fluorescence; and one is based on microfluidic immunofluorescence technology (Table 3). The selection of the Ag-RDT resulted from an expression of interest launched by FIND (www.finddx.org) and a scoring process based on defined criteria. This list includes eight Ag-RDTs on the WHO Emergency Use Listing (WHO-EUL) and six tests that are on the waiting list for WHO-EUL approval (40). Analytical testing was performed on all Ag-RDT brands (Table 3), and a small subset of these was further used for the clinical evaluation on retrospective samples based on brands that showed the best results on clinical diagnostic evaluations under the FIND program (Covios, Hotgen, Onsite, SureStatus) and widely used in the UK for mass testing (Flowflex). Results on prospectively collected samples are only provided with Biocredit.

**TABLE 3:** Overview of the RDT brands used in the study for analytical performance^*a*^

Ag-RDT brand	Test/Company/Country	Target Ag	Principle	Approval
ActiveXpress	ActivXpress+ COVID-19 Ag Complete Kit/Edinburgh Genetics, Ltd./UK	N	G	CE
AllTest	SARS-CoV-2 Antigen Rapid Test/Hangzhou AllTest Biotech, Ltd./China	N	G	CE
Biocredit	Biocredit COVID-19 Ag/Rapidgen, Inc./Rep. of Korea	N	G	CE/WHO EUL
Bioperfectus	SARS-CoV-2 Ag Rapid Test/Jiangsu Bioperfectus Tech., Ltd./China	N	G	CE
Core	COVID-19 Ag Test/Core Technology, Ltd./China	N	G	CE
Covi-Go	Covigo/Mologic, Ltd./UK	N	G	CE/UA
Covios	Covios COVID-19 Ag Test Device/Mologic, Ltd./UK	N	G	CE
Espline	ESPLINE SARS-CoV-2/Fujirebio Diagnostics, Inc./Japan	N	G	CE
Excalibur	Rapid SARS-CoV-2 Antigen Test Card/Excalibur Healthcare Services/UK	N	G	CE
Flowflex	Flowflex SARS-CoV-2 Ag Rapid Test/Acon Biotech, Ltd./China	N	G	CE/ WHO EUL
Fortress	CoV Ag Nasal Swab Rapid Test/Zhejiang Orient Gene Biotech/China	N	G	CE
Genedia	GENEDIA W COVID-19 Ag/Green Cross Medical Sciences/Rep. of Korea	N	G	CE
GeneFinder	COVID-19 Rapid Test GeneFinder/OSANG Health Care/South Korea	N	G	CE/UA
Hotgen	2019-nCoV Antigen Test/Beijing Hotgen Biotech, Ltd./China	N	G	CE
iChroma	iChroma COVID-19 Ag Test/Boditech Medical, Inc./Rep. of Korea	N	F	CE
Innova	Innova SARS-CoV-2 Antigen Rapid/Innova Medical Group, Ltd./UK	N	G	CE/UA
Intec	Rapid SARS-CoV-2 Antigen Test/Intec Products, Inc./China	N	G	CE
Joysbio	SARS-CoV-2 Antigen Rapid Test Kit/Joysbio Biotechnology, Ltd./China	N	G	CE/UA
LumiraDx	LumiraDx SARS-CoV-2 Antigen Test/Lumira Dx, Ltd./US	N	M	CE/ WHO EUL
Nadal	Nadal COVID-19 Ag Test/Nal von minden GmbH/Germany	N	G	CE
NowCheck	NowCheck COVID-19 Ag Test/Bionote, Inc./Rep. of Korea	N	G	CE
Onsite	Onsite COVID-19 Ag Rapid Test/CTKBiotech, Inc./USA	N	G	CE/ WHO EUL
PanBio	Panbio COVID-19 Ag Rapid Test/Abbott Rapid Diagnostics/Rep. of Korea	N	G	CE/WHO EUL
PerkinElmer	PerkinElmer COVID-19 Antigen Test/PerkinElmer/Switzerland	N	G	CE/UA
RespiStrip	Respi-Strip COVID-19 Ag/Coris Bioconcept/Belgium	N	G	CE
RighSign	COVID-19 Ag Rapid Test Cassette/Hangzhou Biotets Biotech, Ltd./China	N	G	CE
Roche	SARS-CoV-2 Rapid Ag Test/Roche Diagnostics/Switzerland	N	G	CE
Standard-F	Standard F COVID-19 Ag FIA., SD Biosensor, Inc./Rep. of Korea	N	F	CE
Standard-Q	Standard Q COVID-19, SD Biosensor, Inc./Rep. of Korea	N	G	CE/WHO EUL
StrongStep	StrongStep SARS-CoV-2 Ag Rapid Test/Nanjing Liming Bio-Products/US	N	G	CE
SureStatus	Sure-Status COVID-19 Antigen Card Test, Premier Medical Corp./India	N	G	WHO EUL
Tigsun	Tingsun COVID-19 Ag Rapid Test/Beijing Tigsun Diagnostics, Ltd./China	N	G	CE
Wantai	Rapid SARS-CoV-2 Antigen Test/Wantai Biological Pharmacy, Ltd./China	N	G	CE
Wondfo	Wondfo 2019-nCoV Antigen Test/Guangzhou Wondfo Biotech/China	N	G	CE/WHO EUL

^*a*^ Ag-RDT: rapid diagnostic test; target N: nucleoprotein; principle G: LFA using gold (colorimetric detection); F: LFA fluoresce detection; M: microfluidic fluorescent technology; CE: CE making as per European conformity; WHO EUL: WHO Emergency Use Listing; UA: under assessment for WHO Emergency Use Listing.

### SARS-CoV-2 viral culture and Ag-RDT limit of detection

The SARS-CoV-2 isolates were grown in Vero E6 cells (C1008; African green monkey kidney cells) and maintained in culture media (Dulbecco’s modified eagle membrane [DMEM] with 2% fetal bovine serum and 0.05 mg/mL gentamycin) as previously described (28, 30). The isolates for Alpha (GenBank accession number: MW980115), Delta (SARS-CoV-2/human/GBR/Liv_273/2021), Gamma (hCoV-19/Japan/TY7-503/2021), Omicron BA.1 (SARS-CoV-2/human/GBR/Liv_1326/2021), and Omicron BA.5 (SARS-CoV-2/South Africa/CERI-KRISP-K040013/2022) were used to evaluate the analytical limit of detection (LOD) of the 34 Ag-RDTs using live virus.

Plaque-forming units per milliliter (PFU/mL) of the viral stocks were counted using viral plaque assay as previously described (29), and 10-fold serial dilutions of the viral stock were made starting from 1.0 × 10^6^ PFU/mL using DMEM as a diluent. Twofold dilutions were made below the 10-fold LOD dilution to determine the LOD. The LOD was defined as the lowest dilution at which all three replicates were positive by Ag-RDT. The LODs for WT, Alpha, and Gamma VOCs obtained as part of our previous work utilizing the same protocol (28, 29) were used here for practicality to compare to the Delta and Omicron lineage LODs.

### Retrospective and prospective clinical samples

**Fig 5:**
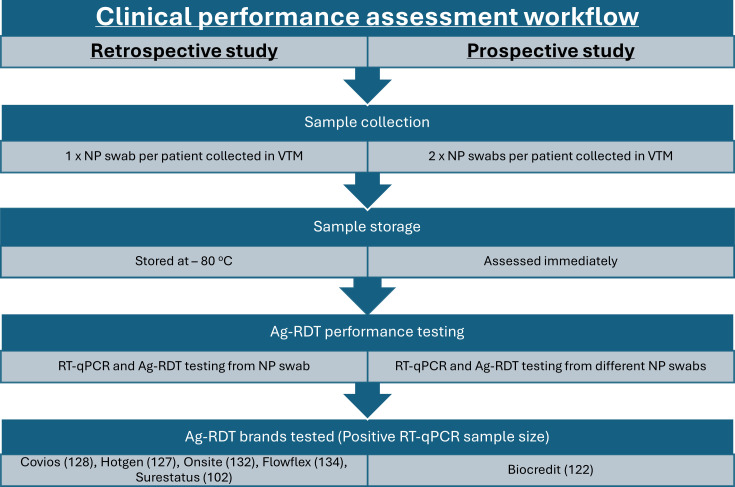
Flowchart comparing the sample collection and Ag-RDT assessment methods in the retrospective and prospective studies. Retrospective study details are on the left-hand side of the chart, while prospective study details are on the right.

Clinical samples were collected as part of the ‘Facilitating Accelerated Clinical Evaluation of Novel Diagnostic Tests for COVID-19’ (FALCON) study (41). Ethical approval was obtained from the National Research Ethics Service and the Health Research Authority (IRAS ID:28422, clinical trial ID: NCT04408170). Nasopharyngeal (NP) swabs in vital transport media (VTM) were collected from consenting symptomatic adults attending the community drive-through COVID-19 test center located in Liverpool John Lennon Airport, UK between January 2021 and March 2022 (Fig. 5). The clinical specimens were transported to the Liverpool School of Tropical Medicine (LSTM) biosafety level 3 (BSL3) laboratories in insulated UN7737 transport bags and aliquoted and stored at −80°C until further testing.

Prospective clinical samples were collected as part of a diagnostic evaluation of the Biocredit Ag-RDT (Table 3) (42). Participants were enrolled from December 2021 to March 2022, coinciding with the emergence of Omicron. NP swabs in VTM were collected for RT-qPCR, followed by another NP swab in the alternate nostril for the Biocredit Ag-RDT evaluation. Specimens were transported to the LSTM BSL3 laboratories as described above and, in this case, processed immediately for Ag-RDT testing.

Samples were confirmed SARS-CoV-2 RNA-positive using the TaqPath COVID-19 CE-IVD RT-PCR Kit (Thermo Fisher Scientific, USA). Based on epidemiological data in the UK at the time of enrollment and S gene amplification in the PCR assay, consecutive SARS-CoV-2-positive RT-PCR samples were selected as presumed Alpha if collected between January and March 2021, presumed Delta if collected between June and August 2021, and presumed Omicron if collected between December 2021 and March 2022. The variant type was later confirmed by whole-genome sequencing. A panel of 10 SARS-CoV-2 RNA-negative VTM samples was also included as negative controls.

### Ag-RDT testing protocol

All Ag-RDTs were performed as specified by the test specific instructions for use (IFU). For the determination of the LOD using live virus, a specific volume of the serial dilutions was added directly to the extraction buffers at a 1:10 ratio as previously described (30). For the clinical samples, VTM was mixed by pipetting at a 1:1 ratio with the extraction buffer of the Ag-RDTs. The dilution factor introduced by the swabs diluted in buffer was accounted for when calculating the viral copy numbers of the tested swab samples. A negative control of only VTM was incorporated to account for any non-specific reaction as previously reported for some Ag-RDT brands when using VTM (30). Results were read by two operators blinded to each other, and, if a discrepant result occurred, a third operator acted as a tiebreaker. The visual readout of the Ag-RDT test band was scored on a quantitative scale from 1 (weak positive) to 10 (strong positive). Ag-RDT results were classified as invalid when the control line was absent.

### Quantification of viral loads

For the quantification of the RNA copy numbers per mL (RNA copies/mL), viral RNA was extracted using QIAmp Viral RNA Mini Kit (Qiagen, Germany) according to the manufacturer’s instructions. The RNA copies/mL were established using the COVID-19 Genesig RT-qPCR Kit (PrimerDesign, UK). RT-qPCR testing was carried out using the Rotor-Gene Q (Qiagen, Germany), with a 10-fold serial dilution of quantified *in vitro*-transcribed RNA incorporated for each PCR run (43). A total of five replicates were tested for each standard curve point, and extracted RNA from each culture dilution was tested in triplicate. The RNA copies/mL for samples were then calculated from the mean Ct value of these replicates.

### Whole-genome sequencing

Clinical samples underwent whole-genome sequencing to confirm the SARS-CoV-2 variant. Sequencing was performed using the ARTIC V3 (LoCost) (44) sequencing protocol on the MinION R.9.4.1 flow cell (Oxford Nanopore Technology, UK). RT-PCR was initially performed with a two-step PCR, the Arctic RT PCR 5× LunaScript RT SuperMix (New England Biolabs, USA) with 8 µL of RNA sample and a thermal cycling profile of 2 min at 25°C, followed by 10 min at 55°C, and then 1 min at 95°C. This was then followed by the Q5 Hot Start High-Fidelity 2× Master Mix (New England Biolabs, USA) using 10 µM of the ARTIC V4.1 primer pools (Integrated DNA Technologies, USA) and a thermal cycling profile of 30 s at 98°C for heat inactivation, followed by 25 cycles of a 15-s denaturation at 98°C and a 5-min annealing/extension at 65°C. Library preparation was carried out using the Ligation Sequencing Kit (SQK-LSK109) and Native Barcoding Expansion Kits (EXP-NBD104 and EXP-NBD114) (Oxford Nanopore Technologies). Basecalling was carried out via MinKnow (v4.2.8), with demultiplexing and read filtering using Guppy (v5.0.7). The ARTIC pipeline was then used to assemble a consensus genome, BAM files, and variant calling file with *--normalise 200 --threads 4*. Variant calling was carried out using EPI2ME Desktop Agent v3.3.0 with the ARTIC+ NextStrain analysis pipeline.

### Statistical analysis

Statistical analyses were performed using SPSS V.28.0, Epi Info V3.01, and R scripts. Binomial confidence intervals for sensitivities and specificities were computed using the Wilson score interval. Differences in the analytical LODs of VOCs were compared using Kruskal-Wallis with Bonferroni correction for multiple tests. To further analyze analytical sensitivities, we used logistic regression, with the RNA copy number as the independent variable and test outcomes as the dependent variable, yielding detection probabilities for each viral load level. A three-way factorial ANOVA was performed on log-transformed genomic Ct values to assess the effects of the RDT result (value: positive vs. negative), variant, and test brand, along with their interactions. Bonferroni-adjusted post-hoc tests were performed to compare RDT+ and RDT− within each variant and test brand.
